# Investigating the Cognitive Basis of Light Training Tasks: The Role of Attention

**DOI:** 10.3390/brainsci16060559

**Published:** 2026-05-25

**Authors:** Marios N. Avraamides, Fotini Hadjivassiliou, Maria Tutuianu, Eirini Katsou, Marigo Georgiou, Andria Shimi

**Affiliations:** 1 Department of Psychology, University of Cyprus, P.O Box 20537, 1678 Nicosia, Cyprusshimi.andria@ucy.ac.cy (A.S.); 2 CYENS Centre of Excellence, 1016 Nicosia, Cyprus

**Keywords:** attention, cognition, reactive agility, sports, exercise, virtual reality

## Abstract

**Background/Objectives:** Light training tasks are widely used in athletic training, yet the cognitive processes that underlie performance on these tasks remain poorly understood. **Method:** In this study, we investigated whether exogenous attentional orienting and visual search abilities could explain individual differences in performance on SpeedPad, a Virtual Reality light training task, in participants who engage in physical exercise and in controls. **Results:** Our results showed that participants engaging in physical exercise outperformed those who did not in SpeedPad sessions that required fast reactions to targets with or without distractors. Furthermore, hierarchical regression analyses revealed that engagement in physical exercise accounted for a significant amount of variance in SpeedPad performance. In addition, both the cueing benefit from an exogenous orienting task and the intercept of a visual search task with feature search trials accounted for unique variance in SpeedPad performance. **Conclusions:** Overall, the current findings suggest that performance in light training tasks such as SpeedPad depends on both physical/physiological and cognitive factors, especially those related to different types of attention.

## 1. Introduction

A critical factor for performance in many sports is the ability to react quickly to an external stimulus. For example, to perform an effective tackle, a defender in soccer must be able to react quickly to the abrupt movement of the opponent. Similarly, a basketball player must respond quickly to the trajectory of the ball to collect a rebound, and so must a volleyball player to recover a ball that is deflected off a block. Thus, in addition to improving physical condition, athletes and their coaches often place much emphasis during training on improving reaction speed to stimuli [1].

Drills aiming to improve reaction speed typically involve presenting abrupt stimuli (e.g., light, sound, movement) to which the athlete is called to react as fast as possible. For example, in whole-body reactive agility tests, the athlete starts running in a specific direction (e.g., forward) and is asked to change direction to the left or the right in response to the location of a sound or a signal from the coach [2,3]. Similarly, in light training tasks, such as the Batak Pro, the Fitlight Trainer system, and SpeedPad, the athlete is called to hit, as fast as possible, targets that light up or change color. Notably, several studies have documented improvements in motor reaction time following training with light training tasks. For example, ref. [4] showed improvements in upper and lower limb reaction times in basketball players following an 8-week training program with the Fitlight Trainer system (see also [5] for similar results with junior players of handball, basketball, and volleyball). Also, ref. [6] showed that basketball players training with Fitlight exhibited greater improvements in dribbling skill and hand reaction than players who completed a similar program without Fitlight training.

In addition, past studies indicate that light training systems and reactive agility tasks with whole-body movement have discriminatory power. For example, ref. [7] showed that handball players had significantly faster reaction times using the Fitlight Trainer than non-athlete controls when carrying out exercises with lights that were placed horizontally in front of them. Also, ref. [8] showed that the extent of engagement in physical activity predicted reaction times in both a simple version of Fitlight that involved responding to target lights with the dominant hand and a complex version that entailed responding with one or the other hand depending on the color of the target light.

In summary, past research documents that (1) athletes have better performance in reactive agility tasks than controls and that (2) training with such tasks improves motor reactions times in the same task used for training [4] or different tasks that involve fast reactions to stimuli [6]. Despite these findings, it is not entirely clear yet what drives either the individual differences in reaction time or the training gains that past studies report. That is, although performance in tasks like this is very likely influenced by physical attributes and neuromuscular efficiency (e.g., balance and stability, motor control, elastic strength), it may also depend on cognitive factors, such as attention, perception, and decision making. Thus, individual differences and/or benefits in motor reaction times might be caused by improvements in physical skill, cognitive factors, or both.

To address the extent to which cognitive factors are involved in carrying out reactive agility tasks, in the current study we examined the discriminatory power of SpeedPad, a flexible light training app that leverages Mixed Reality technology by comparing the performance of participants engaged in physical exercise with that of participants who are not; at the same time, we evaluated participants’ cognitive functioning through traditional cognitive tests. Specifically, we examined whether different attentional processes account for variability in SpeedPad performance.

This study builds upon previous research from our group [9] showing the individual differences in performance on SpeedPad are predicted by attentional orienting (i.e., the ability to orient attention quickly towards stimuli in response to cues) and by split attention (i.e., the ability to divide attention across locations). These findings, along with those from other studies that administered light training tasks along with cognitive tasks such as the D2 Attention Test [8] and the flanker task [9], suggest that the efficient deployment of attention is critical for high performance in these training tasks. Given that attention is multifaceted, the present study extends the literature by assessing different attentional processes to those studied before, addressing at the same time limitations in past research. For example, in the study of [10], SpeedPad performance was predicted by reaction time in a Posner cueing task but not by the cueing effect (valid–invalid RT difference). This suggests SpeedPad captures attentional orienting speed but not the ability to strategically use cues. A limitation was that the cuing task used measured top-down (endogenous) orienting, whereas SpeedPad likely relies more on automatic, bottom-up (exogenous) attention. Similarly, visual search efficiency also correlated with SpeedPad but did not explain unique variance. However, the visual search task used conjunction search, which is effortful and serial, unlike SpeedPad’s pop-out (feature search) demands.

Thus, in the current study, we used an exogenous cueing task, included both feature and conjunction search trials, added distractor lights in SpeedPad, and increased sample size to better test group differences. This allowed us to explore through regression analyses whether the automatic orienting of attention and the efficiency of searching for a target among distractors would explain individual differences in SpeedPad performance. In addition, we further examined individual differences in SpeedPad performance and cognitive function by dividing participants in two groups: those who reported engagement in physical activity vs. those who did not.

Our main prediction, based on the findings of [7] and Reigal et al. [8], was that participants who engage in exercise would demonstrate better performance in SpeedPad. Based on past studies documenting the involvement of cognitive skills in sports (e.g., [11]; for reviews on the topic see also [12,13]), we also expected participants who engaged in physical activity to outperform those who did not in the two attentional tasks (exogenous cueing, visual search) as well. We also hypothesized that participants with faster attentional orienting in response to cueing would perform better on a simple scenario of SpeedPad without distractors, clearly implicating exogenous attentional capture. Of interest was to see whether this would also be the case in a more complex scenario of SpeedPad, which requires filtering out distractors and might thus implicate top-down processes such as serial visual search. Therefore, we also expected visual search efficiency in the conjunction search trials to predict individual differences in the complex scenario of SpeedPad that involved distractors but not in the simple scenario. Of interest was to examine whether attentional orienting and visual search would explain unique variance in SpeedPad performance beyond that explained by engagement in physical activity. Such a result would provide convincing evidence that light training tasks such as SpeedPad rely, in addition to physical skills, on cognitive functions as well.

Identifying the specific cognitive abilities engaged during light training tasks such as SpeedPad will help determine whether these tasks can produce transferable training benefits for athletic activities in which those cognitive abilities are critical. This long-term objective is motivated by the growing evidence showing that immersive VR training tasks that engage cognitive processes can lead to both short- and long-term cognitive benefits (e.g., [14,15]).

## 2. Method

### 2.1. Participants

A power analysis using G*Power v.3.1.96 [16] revealed that, for powering the entire model of a stepwise regression (i.e., a stricter criterion than powering each incremental step), 59 participants were needed to detect a medium-to-large effect size f^2^ = 0.20 with 1 predictor in each of 3 steps, assuming α = 0.05 and power = 0.80. Thus, we recruited 70 healthy adult participants between 18 and 35 years of age (mean = 20.6, SD = 3.06) to participate in the study. Of these, 60 were women. Participants were recruited through the undergraduate and graduate community of the University of Cyprus and the community. Exclusion criteria included a history of neurological disorders and any self-reported motor impairments or movement difficulties. Students enrolled in courses were given course credit in exchange for their participation. Participants were divided into the exercise and the non-exercise groups based on information they provided about their exercise habits and participation in sports (i.e., whether they were currently or in the past 2 years engaged in regular sport activity, either at a professional or at amateur level). Based on their responses, 38 participants comprised the Exercise Group and 32 participants the Non-Exercise Group. Those in the Exercise Group were participants that reported playing sports or dancing on a regular basis (at least 3 times per week) within the last 2 years. Specifically, 5 participants in the Exercise Group reported regular sport participation in the recent past, 14 individuals reported regularly going to the gym, 3 partook in Martial Arts, 9 were involved in different types of Dancing, and the remaining 7 each participated in one of the following: Swimming, Volleyball, Tennis, Rowing, Football, Badminton, and Track. Participants in the No Exercise group reported neither current engagement in sport/dancing nor exercise. Also, none of them reported engagement in sports or physical activity in the last 2 years. All participants read and signed an informed consent form prior to their participation. The study protocol was reviewed and approved by the Cyprus National Bioethics Committee.

### 2.2. Materials

A cueing task and a visual search task were developed and administered in OpenSesame v.4.0 [17] to evaluate distinct attentional processes. SpeedPad, a commercial Augmented and Virtual Reality (VR/AR) app, was used to assess reactive agility.

**Figure 1:**
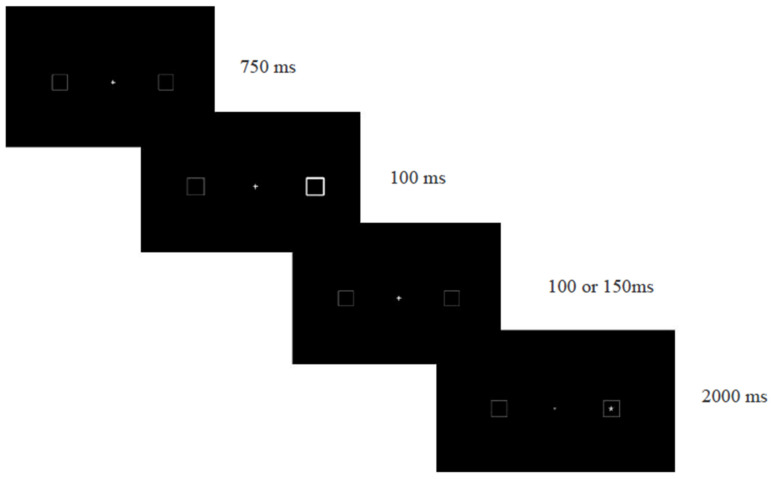
Example of a valid cue trial in the Posner Exogenous Orienting task. The target asterisk appears in the correctly cued location, indicated by the bold outline of the square.

**Posner exogenous cueing task.** Participants responded to a target following valid or invalid non-informative peripheral cues. Thus, it measured how quickly attention was oriented automatically to the cued location (valid trials) and how quickly participants disengaged attention from the cued location and voluntarily moved it to the alternative location (invalid trials). In this task (Figure 1), participants were first presented with a fixation cross in the center of the display, flanked by two potential target locations, i.e., two squares, one on the left and another on the right side. Following a short delay, one of the two locations was cued to draw the participant’s attention there. Peripheral cues were outline borders that became thicker when that location was cued. After another short delay, a target stimulus (an asterisk) appeared either at the cued location (valid trial) or at the opposite, non-cued location (invalid trial). Participants pressed, as quickly as possible, a key to indicate the side (left or right) in which the target appeared.

**Figure 2:**
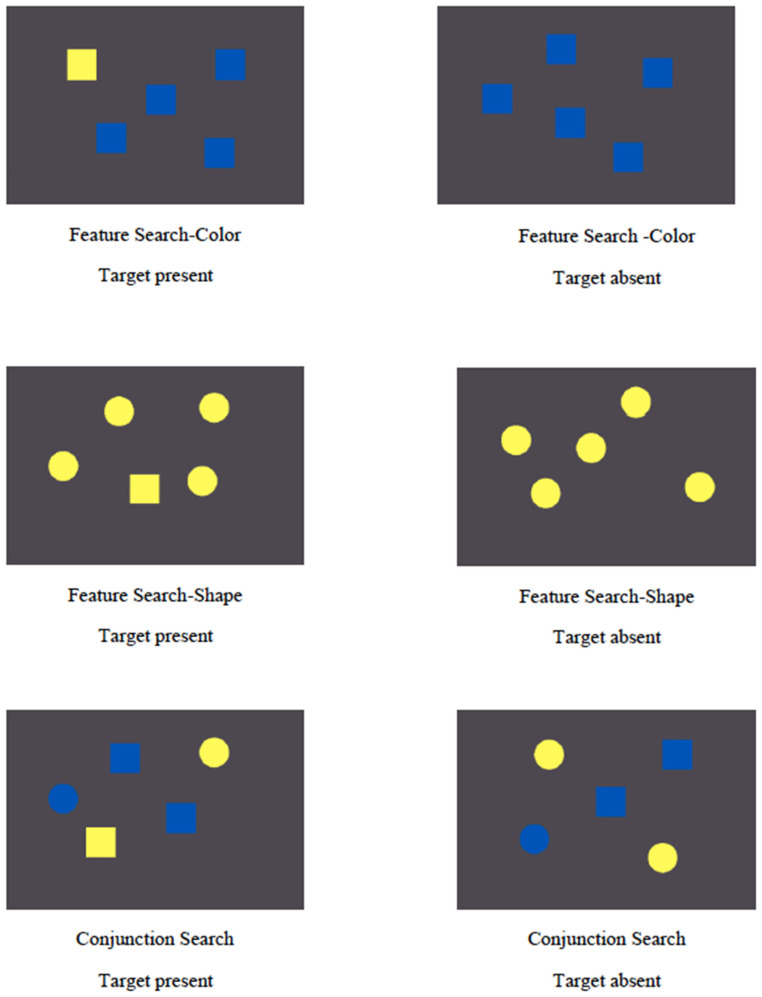
Visual search examples: feature and conjunction tasks with 5 items in the display and a yellow square as the target.

**Visual Search Task.** The visual search task assessed how efficiently participants located a target presented in a display among a set of distractors (Figure 2). They were presented with displays containing varying numbers of items and determined whether a specified target was present or absent in the display and pressed a corresponding key (X for present, Y for absent). Depending on the similarity between target and distractors, the task included trials of feature or conjunction search. In feature search trials, the target differed from distractors in a singular unique feature like color, shape, orientation, or size. An example of a feature search is looking for a red X among black X’s. In contrast, a conjunction search occurred when the target was defined by a combination of features that were also present in distractors, e.g., looking for a red X among red T’s and black X’s. In the current version, participants were presented with displays containing 1, 5, or 15 items (including the target), with blue or yellow squares and circles. The task included both feature and conjunction search trials. In the feature search trials, the target could be, for example, a yellow square presented among blue squares and circles (color feature), a blue square presented among blue circles (shape feature), and so on. In the conjunction search trials, the target could be, for example, a yellow circle presented among yellow squares and blue circles, defined thus by a combination of the features (i.e., color and shape) that were also present in distractors. For both types of trials, participants had to press the left arrow key if the target was present and the right arrow key if the target was absent.

**Figure 3:**
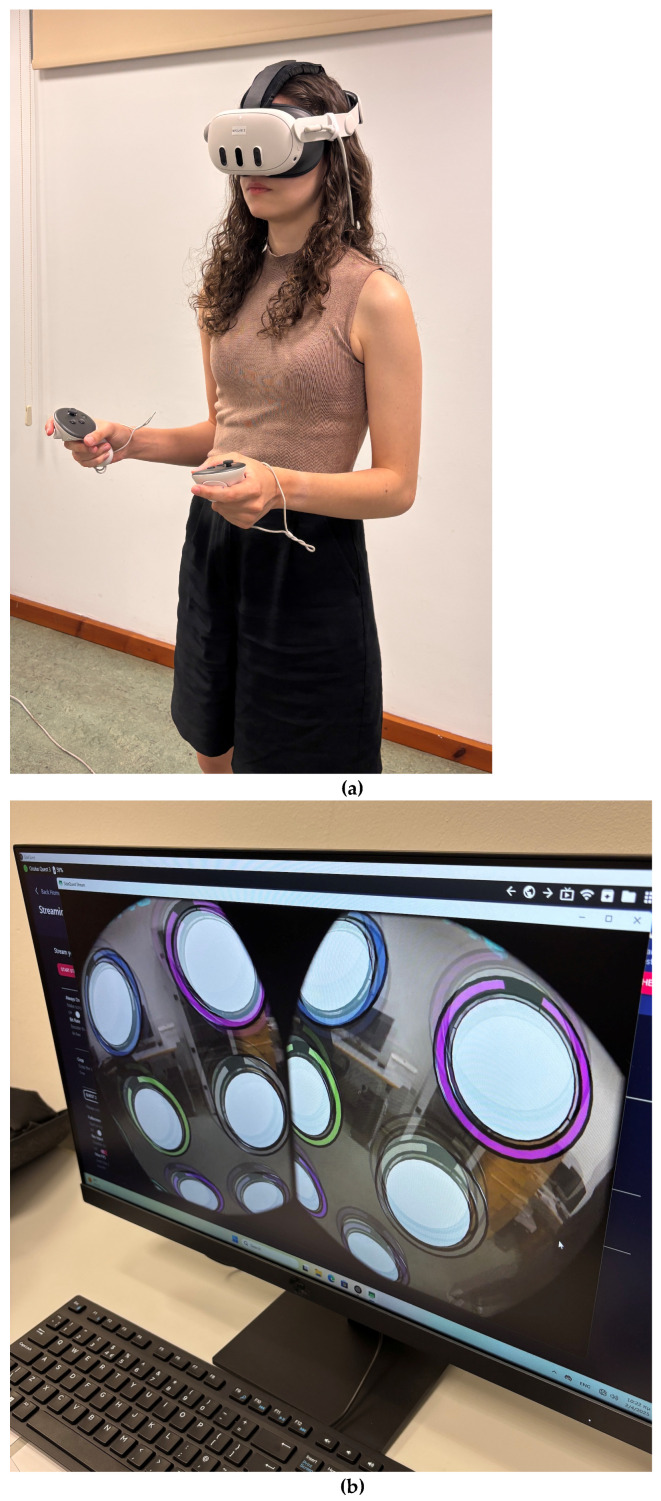
(**a**) participant wearing the VR headset. (**b**) View of SpeedPad streamed to a computer screen. Participants had to touch the disc whose periphery became red and ignore the other discs.

**SpeedPad.** To assess reactive agility, we used a commercial VR/AR app called SpeedPad (MentisVR Ltd, Nicosia, Cyprus). When donning a VR headset, participants are presented with an array of white discs presented either within the physical environment (AR mode) or within a selection of virtual environments (VR mode). When the task starts, discs turn red in a random order, and participants reach out with their arms to touch each disc that turns red (Figure 3). Virtual hands are mapped onto the handheld controllers, allowing participants to touch the surface of each disc. The number of red discs touched within a set period is recorded and can be used as an index of the performance. Task complexity is varied by setting the number of discs in the array, the speed and frequency in which they turn red, whether distractor discs change to other colors along with the target turning red, etc. For the present study we used a 9-disc array in AR mode. We set up two scenarios, one to measure *Simple* accuracy and another to measure *Complex* accuracy. In the Simple condition, the target disc turned red while all other discs remained white. In the Complex condition, when the target disc turned red, all other discs changed as well to other colors. For both conditions, we measured the number of hits (the number of target discs touched correctly) and the number of false alarms (the number of distractors erroneously touched) within 1 min.

### 2.3. Procedure

Participants were tested individually in a quiet laboratory at the University of Cyprus in a 1 h session. Before participation, they read and signed informed consent forms. The tasks were administered in the same fixed order for all participants. SpeedPad was presented last to prevent potential carry-over of physical fatigue into the cognitive assessments. Pilot testing with the computerized tasks showed that performance on the two tasks did not differ as a function of administration order. Nevertheless, participants were allowed to take breaks of any duration they wished in order to minimize fatigue.

First, participants carried out the Posner cueing task. They were seated in front of a computer screen and were instructed to rest their index fingers on the F and J keyboard keys. Each trial started with a central fixation cross that appeared on the display for 750 ms, along with the two squares indicating the possible locations in which the target could appear. Following a delay of 750 ms, the outline of one of the two squares became thicker for 100 ms, cueing that particular location. Following an interstimulus interval of either 100 or 150 ms, the target was presented. The target was an asterisk that was presented in one of the two squares and remained on screen until the participant entered a response, up to a maximum of 2000 ms. The participants were asked to respond quickly and accurately by pressing the F key if the asterisk appeared in the left square or the J key if it appeared in the right square. Feedback was then presented to participants by presenting a fixation cross for 200 ms either in blue color for correct responses or red color for incorrect responses. Trials with valid and invalid cues were presented randomly and in the same proportion across the task. Participants completed a total of 192 trials (6 blocks of 32 trials each) and were given feedback for the accuracy and reaction time. Prior to the experimental trials, participants carried out 8 practice trials to familiarize themselves with the task.

Following the cueing task, participants carried out the visual search task. For this task, participants were asked to rest their index fingers on the left and right arrow keys. Each trial of the task started by specifying the target, which could be a yellow circle, a blue circle, a yellow square, or a blue square. Then, a display containing 1, 5, or 15 shapes was presented. Participants were instructed to search for the target in the display and respond as fast as possible by pressing the left arrow if the target was present in the display or the right arrow if the target was absent. The search display remained visible until they responded, up to a maximum of 2000 ms. Participants completed a total of 288 trials, divided across 4 blocks. Each block contained an equal number of feature search trials in which the target differed from the distractors with respect to a single feature, shape or color and conjunction search trials in which the target was defined by a combination of features that were also present in the distractors. Prior to the experimental trials, participants carried out 18 practice trials, 9 with a feature search and 9 with a conjunction search.

Finally, following the completion of the visual search task, participants were asked to perform two tasks in the SpeedPad app. To this purpose, they stood in the middle of the room and were fitted with a Meta Quest 3 VR headset. They were also handed the Quest controllers to hold in each hand. After a practice session in which participants became familiar with the tasks, they proceeded to carry out the Simple scenario. In this scenario they punched with the controllers each disc that turned red in an array of 9 white discs, which was presented in front of them as a 3 × 3 matrix. Participants performed the tasks for 1 min and were instructed to respond as fast as possible to the target and avoid punching distractors. Once the minute elapsed, a table was presented in the headset displaying the number of hits, misses, correct rejections, and false alarms for the session. The experimenter copied these scores on a hard-copy response sheet. Once participants completed the Simple scenario, they took a short resting break, and when they were ready they proceeded to the Complex scenario. Everything in the Complex scenario was identical to the Simple one, with the exception that when the target disc turned red, all other discs changed to different colors. In both conditions, the target remained red until it was hit or until a set timer of 7 s ran out. Throughout the VR task, the display of the headset was cast to a computer screen so that the experimenter could monitor task execution.

## 3. Statistical Analyses

First, we examined whether the cueing task and the visual search tasks produced the pattern of results expected in the literature, and then we carried out analyses to determine performance differences across participants who exercised vs. those who did not.

For the cueing task, we used a *t*-test to examine whether responses were faster for valid compared to invalid trials, yielding the expected cueing benefit. To examine whether there were differences in reaction times for valid and invalid trials across the exercise and non-exercise groups, we then conducted a repeated-measures Analysis of Variance (ANOVA) with Trial Type (Valid vs. Invalid Trials) as a within-participants variable and Group (Exercise vs. Non-Exercise) as the between-participants variable.

To verify that the visual search task we used replicated the expected pattern of results documented in the literature, we carried out a repeated-measures ANOVA with terms for Search Type (Feature search vs. Conjunction search) and Set Size (1, 5, 15) for the participants’ reaction times (RTs). Then, we used a custom script in R to compute the RT x set size function and extract each participant’s intercept and slope values. Intercept values index participants’ baseline processing speed, which is essentially the time it takes them to make a decision while unaffected by distractors. Slope values index the effect of each distractor in the display on participants’ search speed. Thus, a small slope value translates to high search efficiency as it indicates that the participants were not greatly influenced by the distractors. In contrast, large slope values index low search efficiency, as they indicate a large effect of distractors on search speed. Following this computation, we carried out separate repeated-measures ANOVAs to compare intercepts and slopes for feature and conjunction search trials between the exercise and the non-exercise groups.

For SpeedPad, we carried out a repeated-measures ANOVA on the number of hits with terms for Scenario Type (Simple vs. Complex) and Group (Exercise vs. Non-Exercise).

We followed-up the measure-specific analyses with correlational analyses to investigate possible relations between the computerized tasks and SpeedPad. Finally, to investigate whether individual differences in SpeedPad could be explained by our cognitive measures, we performed a hierarchical linear regression for each of the Simple and the Complex sessions of SpeedPad, using the engagement in physical exercise and one RT measure from each of the computerized tasks as predictors. Based on observation of the simple correlations, we selected the cueing benefit and the intercept value of feature search trials as predictors.

## 4. Results

### 4.1. Cueing Task

**Figure 4:**
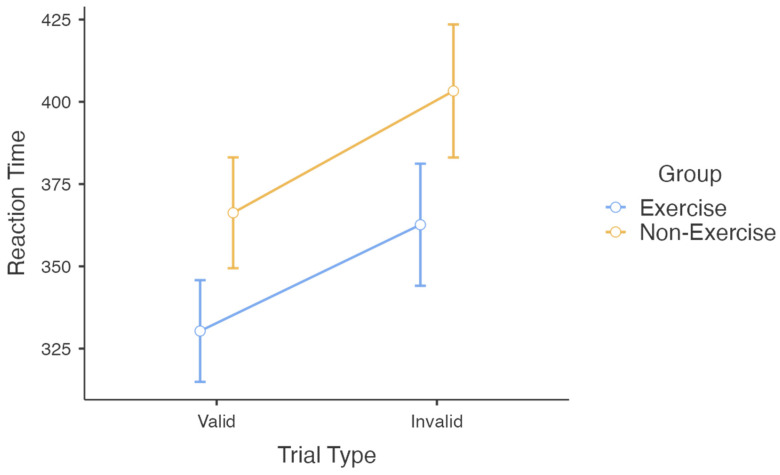
Comparison of reaction times between Exercise and Non-Exercise groups across trial types.

A paired-sample *t*-test revealed the presence of a cue benefit, with response times being shorter for valid cue trials (M = 347 ms) than invalid cue trials (M = 381 ms), t(69) = 10.5, *p* < 0.001. This finding verifies that the exogenous cueing task used here yielded the expected pattern of results as documented in the literature [18]. A repeated-measures ANOVA with terms for Trial Type and Group showed that in addition to the main effect for the Trial Type F(1,68) = 110.26, *p* < 0.001, η^2^_p_ = 0.62), there was a significant main effect for Group, F(1,68) = 9.82, *p* = 0.003, η^2^_p_ = 0.13. As seen in Figure 4, the Exercise Group was faster than the Non-Exercise Group in both valid and invalid trials. The interaction between Trial Type and Group was not significant, F(1,68) = 0.51, *p* = 0.48.

### 4.2. Visual Search

**Figure 5:**
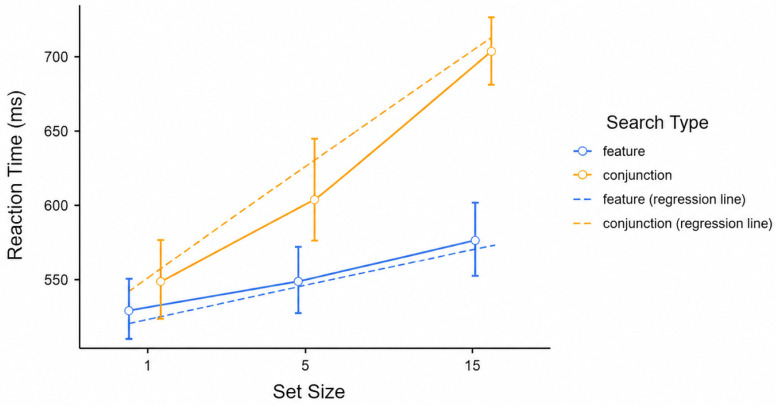
Effect of set size on reaction time for feature and conjunction searches.

A repeated-measures ANOVA with terms for Search Type (Feature search vs. Conjunction search) and Set Size (1, 5, 15) for the participants’ RTs revealed a highly significant main effect of Search Type on RT, F(1,69) = 153.5, *p* < 0.001, η^2^_p_ = 0.69. The RT for Feature searches was significantly shorter (M = 554 ms) than for Conjunction searches (M = 619.7 ms, t = −12.4, *p* < 0.001). Furthermore, the main effect of Set Size on RT was also highly significant, F(2,138) = 133.3, *p* < 0.001, η^2^_p_ = 0.66. Post hoc tests indicated significant differences between all pairs of set size: 1 vs. 5 (t = −6.02, *p* < 0.001), 1 vs. 15 (t = −17.68, *p* < 0.001), and 5 vs. 15 (t = −9.36, *p* < 0.001). More importantly, a significant interaction between Search Type and Set Size was found, F(2,138) = 38.7, *p* < 0.001, η^2^_p_ = 0.36. As seen in Figure 5, the interaction was due to the more dramatic increase of RT with set size in conjunction compared to feature search trials; this finding is fully consistent with the previous literature on visual searches [19].

To examine potential differences in visual search performance across the Exercise and Non-Exercise groups, we carried out separate repeated-measures ANOVAs to compare intercepts and slopes for feature and conjunction search trials between the exercise and the non-exercise groups.

The analysis for intercepts revealed a marginal effect for Search Type, F(1,68) = 3.49, *p* = 0.066, η^2^_p_ = 0.05. The mean intercept value across participants was higher for Conjunction (M = 546 m) than Feature Search trials (M = 533 ms). Neither the main effect of Group nor the Group × Search Type interaction was significant, F(1,68) = 0.15, *p* = 0.70 and F(1,68) = 1.50, *p* = 0.23, respectively.

The analysis for the slopes revealed a significant main effect for Search Type, with slope values being higher for the conjunction search (M = 10.55) than the feature search (M = 3.12 ms), F(1,68) = 80.93, *p* < 0.001, η^2^_p_ = 0.54. As with intercepts, neither the main effect of Group nor the Group × Search Type interaction was significant, F(1,68) = 1.06, *p* = 0.31 and F(1,68) = 0.40, *p* = 0.53.

### 4.3. SpeedPad

**Figure 6:**
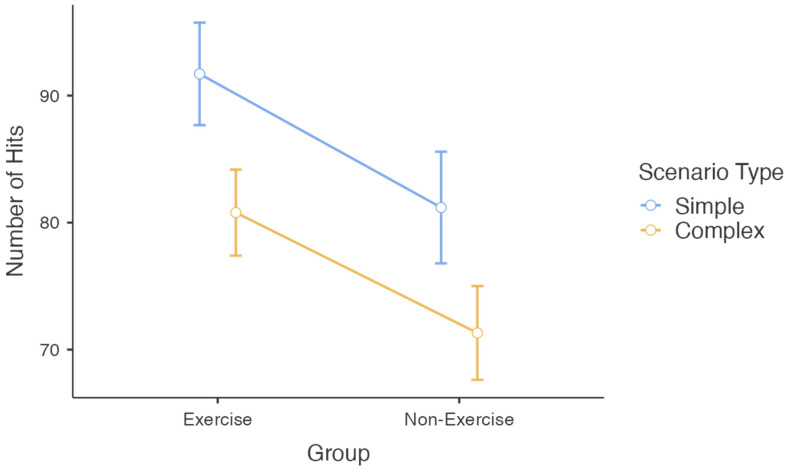
Comparison of number of hits on SpeedPad between Exercise and Non-Exercise groups: Simple vs. Complex scenarios.

We carried out a repeated-measures ANOVA on the number of hits with terms for Scenario Type (Simple vs. Complex) and Group (Exercise vs. Non-Exercise). The results revealed more hits in the Simple (M = 86.4) than in the Complex Scenario (M = 76.1), F(1,68) = 148.84, *p* < 0.001, η^2^_p_ = 0.69. Moreover, as seen in Figure 6, the Exercise Group had significantly more hits (M = 86.3) than the Non-Exercise Group (M = 76.3), F(1,68) = 14.5, *p* < 0.001, η^2^_p_ = 0.18. As seen in Figure 6, the interaction between Scenario Type and Group was not significant, F(1,68) = 0.38, *p* = 0.54. The number of False Alarms was very low (M = 0.86) and did not differ across Group or Scenario Type. Thus, False Alarms were not considered further.

Correlations. To investigate possible relations between the computerized tasks and SpeedPad, we carried out correlation analyses. First, an initial analysis revealed a significant correlation between the Simple and Complex task in SpeedPad, r(68) = −0.851, *p* < 0.001. For the cueing task, the analyses revealed significant negative correlations between the number of hits in SpeedPad (in both the Simple and the Complex task) and the RT for valid and invalid cues in the cueing task (Table 1). More importantly, performance in SpeedPad correlated negatively with cueing benefit, i.e., the difference in RT between invalid and valid trials, r(68) = −0.63, *p* < 0.001 and r(68) = −0.49, *p* < 0.001, respectively, for the Simple and Complex tasks.

Furthermore, we investigated the associations between visual search performance and SpeedPad scores. As shown in Table 2, we found negative correlations between the intercept of visual search for both feature and conjunction trials, and the number of hits in both the Simple and Complex SpeedPad tasks. However, the slope of the visual search function did not correlate with SpeedPad performance.

Linear Regression. Finally, to investigate how much of the variance in the SpeedPad task can be explained by our cognitive measures, we performed a hierarchical linear regression for each of the Simple and the Complex sessions of the task. Three models were tested to determine the explanatory power and significance of these predictors. Model 1 included exercise as a predictor, Model 2 added Cueing Benefit, and finally Model 3 added the intercept value of feature search trials in the visual search task. Notably, although the intercept value of the conjunction search correlated significantly with both the Simple and Complex score in SpeedPad, adding it as predictor in Model 3 did not yield a significant coefficient. Thus, we added only the intercept of feature search in the models we reported.

For the Simple task, whether participants exercised or not explained 15.4% of the variance (R^2^ = 0.154, *p* < 0.001). Adding Cueing Benefit in Model 2 increased the explained variance to 43.9%, R^2^ = 0.439, with a significant change, ΔR^2^ = 0.28, F(1,67) = 34.06, *p* < 0.001. Adding the intercept of feature search in Model 3 explained 47.2% of the variance, R^2^ = 0.472, with a significant change, ΔR^2^ = 0.03, F(1,66) = 4.03, *p* = 0.049. Table 3 displays the model coefficients for the Simple task in SpeedPad.

Results were similar for the Complex task. Model 1, which only included exercise, explained 17.3% of the variance (R^2^ = 0.173, *p* < 0.001). With the addition of Cueing Benefit in Model 2, 37.5% of the variance was explained (R^2^ = 0.375, *p* < 0.001), with the change being significant (ΔR^2^ = 0.20, F(1,67) = 21.69, *p* < 0.001). Finally, with the inclusion of the feature search intercept in Model 3, the explained variance increased to 41.6% (R^2^ = 0.416, *p* = 0.037), with a significant change (ΔR^2^ = 0.04, F(1,66) = 4.55, *p* = 0.037). Model coefficients for the Complex task in SpeedPad are presented in Table 4.

## 5. Discussion

The present study aimed to assess the discriminatory power of light training tasks and investigate the attentional subprocesses they rely on. In respect to discriminatory power, we found that participants who reported current and past engagement in sports and dancing activities outperformed those who did not in both the simple and the complex scenarios of SpeedPad. This finding is aligned with the finding of [7] that handball players do better than controls in light training tasks, as well as with the results of [8], showing that engagement in physical activity predicts performance.

In addition, participants who reported engagement in physical activity were faster than those who did not in the exogenous cueing task in trials with both valid and invalid cues. Given the automatic nature of this task, this finding suggests that participants engaged in physical activity had sharper reflexes, orienting their attention faster towards a validly cued location. Notably, that the advantage was present in invalid trials as well indicates that they were also more efficient in turning their attention away from a location that provides no benefit. Thus, our findings with the cueing task document that participants engaged in exercise had a more efficient attentional disengagement and re-orienting allocation mechanism were in line with the findings of [10], which used endogenous orienting.

The advantage of participants reporting engagement in physical activity in both SpeedPad and the exogenous cueing task is in line with the conclusions of the meta-analyses of [12] and [13]: that performance in cognitive tests varies with sport expertise. First, ref. [12] reported a small-to-medium-sized effect for the overall athlete effect, with athletes performing better than controls in measures of processing speed and some types of attention. Then, ref. [13] reported a medium effect size for the difference between overall cognitive function and skills between higher and lower-skilled athletes. Notably, the effects were larger for tasks that used sport-specific stimuli compared to general stimuli. Despite both SpeedPad and the exogenous cueing task using general stimuli and our sample not including professional athletes, we found significant effects of engagement in physical activity. The difference being present in the cueing task, a task that does not require much physical effort, suggests that the advantage of participants reporting engagement in physical activity was cognitive or in addition to physical.

Importantly, the efficiency of the attentional orienting system, as indexed by the cueing benefit in this task, was a significant predictor of SpeedPad performance over and above physical engagement. Thus, overall, our results suggest that athletes may have more advanced attentional control (including attentional orienting, disengagement, and re-allocation of attention), a cognitive mechanism that underlies performance in SpeedPad along with other attributes that may develop from physical engagement, e.g., physical endurance. As in this study we did not measure any physical attributes, it remains to be seen in future research what these attributes are.

In addition to attentional orienting, we expected participants who engaged in physical activities to also have more advanced visual search skills and that these skills would also predict SpeedPad performance. Our findings did not provide evidence of that. First, in both feature and conjunction search trials, participants engaged in exercise had similar performance to those who did not. Second, the slope of the visual search functions did not correlate with SpeedPad performance. That said, the intercept in the visual search functions did correlate with SpeedPad performance and also predicted unique variance in performance over and above engagement in physical activity and attentional control. However, the intercept in visual search indexes the time required to detect a stimulus in the absence of distractors. Thus, it can be regarded more as an index of detection speed rather than an index of visual search capacity.

Taken together, our findings from the two tasks indicate that SpeedPad relies on three cognitive factors: the ability to detect information quickly, the ability to orient attention reflexively to a location in space, and the ability to disengage attention and quickly re-orient to a new location, after it has been reflectively directed to a location in space. Current results also indicate that people engaged in physical activities may have more-developed orienting and re-orienting abilities than those who do not exercise.

The finding of a group effect in SpeedPad and the cueing task but not in the visual search task is intriguing. One possible explanation relates to the different demands of the tasks. While SpeedPad and the cueing task entail the anticipation of targets that appear suddenly, visual search is more about scanning the visual field for a target that is already there. Although scanning is a common activity in many sports, it may rely on core cognitive processes that do not develop much with practice. Indeed, past studies document that although overall reaction time in visual search tasks can be reduced with practice, the set-size slopes indexing search efficiency do not change [20,21]). Perhaps then, our participants who carried out 288 trials of visual search had adequate practice to reach their maximum search speed, masking any initial differences from sport engagement.

In the context of sports, past research suggests that in terms of visual search behaviors, athletes and non-athletes differ qualitatively rather than quantitatively. For example, a meta-analysis by [22] concluded that, in sport-specific visual search tasks, athletes carry out fewer but longer fixations, as well as longer quiet eye periods, indicative of more efficient visual information sampling. The same meta-analysis concluded that differences in reaction times between athletes and non-athletes are generally small and inconsistent and that athlete advantages are primarily linked to anticipation (see also [23,24] for empirical evidence). The involvement or not of anticipation could explain why in the present study we found group differences in the SpeedPad and the cueing task but not in the visual search task. That said, it should be noted that the intercept of the visual search function correlated negatively with SpeedPad performance and contributed unique variance in the regression analyses. Our conjecture is that these results reflected general processing speed rather than visual search efficiency.

Overall, our findings complement past studies aiming to understand the cognitive processes that underlie light training and reactive agility tasks in general. In our view, this knowledge is essential for understanding what drives the improvements that are documented in these tasks. For example, as noted in the Introduction, ref. [6] showed that basketball players training with Fitlight exhibited greater improvements in dribbling skill and hand reaction than players who completed a similar program without Fitlight training. Yet, it is not clear what drove these gains. Was it an improvement in physical skills such as motor control or elastic strength that led to better dribbling skill and shorter hand reaction time? Or was it an improvement in cognitive processes such as attentional control that allowed participants to orient and re-orient their attention more efficiently? While our findings cannot speak about the real cause of the improvement reported in [6] or other similar studies (e.g., [4]), they do suggest that improvement may indeed be due, at least partly, to cognitive factors relating to attention and overall processing speed. Future training studies with such reactive agility tasks, looking also at how perception and attention might change with training, might shed more light on this topic.

Obtaining a nuanced understanding about cognitive processes that underlie light training and reactive agility tasks will allow using these tools in a more targeted and personalized manner by athletes and their coaches. For example, knowing that light training tasks such as those in SpeedPad rely on the fast detection of target stimuli and on the efficient attentional orienting subprocesses allows one to use the task more confidently with athletes whose sport or position involves these cognitive processes. For example, SpeedPad seems more suitable for a soccer goalkeeper whose main job is to anticipate and react to shots than a midfielder whose job may rely more heavily on other processes such as visual search.

Thus, in our view, effective cognitive training tasks must engage the same cognitive processes required by the target activity. Previous research using immersive VR has produced promising findings when training interventions closely matched the cognitive demands of the outcome measures. For example, a randomized controlled trial with e-sport athletes by [14] found that training with Beat Saber, an immersive VR rhythm game in which players use sabers to slash colored blocks synchronized with music, improved performance on measures of concentration and alternating attention. These benefits likely emerged because the game places high demands on sustained focus and rapid attentional switching, requiring players to continuously identify block colors and respond with the corresponding saber under time pressure.

Although our study provides important new insights about light training tasks, we must acknowledge two important limitations regarding our sample. First, the study recruited participants who engaged in physical activity, a selection criterion that was defined rather loosely as “any type of physical activity undertaken currently or in the near past”. As a result, we ultimately had a diverse experimental group that included participants engaging in different sports as well as dancing, for various periods of time. While this was done on purpose to avoid focusing the study on a particular sport and expertise level, this means that we grouped together participants engaging in activities that may differ substantially in terms of attentional and perceptual demands as well as in spatial ability, e.g., dancing vs. martial arts. Thus, it is highly important that future studies investigate more uniform groups (e.g., soccer players) or even specific sub-groups (e.g., soccer goalkeepers). Of interest would be the examination of whether light training tasks can discriminate not just athletes from non-athletes but also athletes in the same sport but at different levels of expertise, e.g., elite vs. amateur soccer players. Second, the majority of our participants were female, with only 10 male participants included in the sample. Although this differs from much of the existing sports literature, which has historically focused predominantly on male athletes, we believe this represents an important and underrepresented perspective in the field. At the same time, the gender distribution of our sample may limit the generalizability of the findings across broader athletic populations. Importantly, however, including gender as a factor in our statistical analyses did not meaningfully alter the overall pattern of results, suggesting that the observed effects were relatively robust across male and female participants. Another limitation of the current study is that we did not assess any variables related to motor control that could account for individual differences in SpeedPad performance. Future studies may explore whether variables related to online motor control efficiency and stability (e.g., jerk) may explain additional variance in reactive agility tests tasks.

Despite these limitations, the current findings further our knowledge about the role of cognition in light training tasks that are commonly used by athletes in various sports despite the limited knowledge we have about of what they train. Specifically, our results indicate that good performance in light training tasks such as SpeedPad requires fast stimulus detection and efficient control of attention. As both skills are important in various sports, our results align with the past literature, suggesting that light training tasks could potentially be useful tools for exercising aspects of attention and improving processing speed in athletes. The current findings may also help explain why SpeedPad performance has previously been shown to correlate with performance in a virtual goalkeeping task [25]. Presumably, similar to SpeedPad, successful shot-blocking as a goalkeeper relies on rapid stimulus detection and efficient attentional control.

Coupled with the findings of [10], the results from the present study provide a clearer picture about the cognitive processes that underlie light training tasks. These results can serve as the basis for new intervention studies with light training that will target processes and tasks that rely on the control of attention. Although a few past studies have already used light training as an intervention with positive results (e.g., [4,5,6]), it is yet not clear how exactly the benefits came about. To this end, our findings may offer some insights. For example, in the studies by [4,5,6], the benefits were present in outcome measures that required the fast orienting of attention to target stimuli. Based on the current findings, we may posit that Fitlight training in these studies boosted attentional orienting abilities. More importantly, our findings may explain the lack of benefits reported in other studies. For example, ref. [26] found no differences in cognitive function and physical fitness between a group of male soccer players aged 10–15 that trained with Fitlight vs. a control group. Notably, in this study cognitive function was assessed using a Figure Drawing test and a Pen-to-Point test. Neither of these tests involves attentional control. In sum, our findings suggest that future studies using light training tasks as interventions should look for training benefits in tasks that rely heavily on the fast orienting of attention to stimuli.

## 6. Conclusions

The present study contributes valuable new results in the growing literature on the cognition of sports [27,28,29]. Our findings suggest that attentional processes are important for tasks that require fast responses to stimuli and can thus represent important targets for training interventions. In general, the present study advances our understanding about a task that is commonly used in sports, contributing important knowledge about the cognitive processes that could serve in the future as targets for training and improvement. In addition, by corroborating the involvement of cognitive processes in SpeedPad, the results also suggest that VR can serve as a useful medium for administering cognitive training interventions for different sports (see ref. [30] for a review of interventions using VR in soccer). In contrast to training with physical equipment, VR generally provides more flexibility by allowing training to take place anywhere and at any time while providing sport-specific context as needed.

## Figures and Tables

**Table 1:** Correlations between SpeedPad (SP) performance and reaction time measures from the cueing task.

		SP Simple Hits		SP Complex Hits	
Valid	Pearson’s r	−0.353	*	−0.391	**
	df	68		68	
	*p*-value	0.018		0.005	
Invalid	Pearson’s r	−0.553	***	−0.548	***
	df	68		68	
	*p*-value	<0.001		<0.001	
Cueing Benefit	Pearson’s r	−0.566	***	−0.484	***
	df	68		68	
	*p*-value	<0.001		<0.001	

Note. * *p* < 0.05, ** *p* < 0.01, *** *p* < 0.001. *p*-values have been adjusted for multiple comparisons using a Bonferroni correction for 6 tests.

**Table 2:** Correlations between SpeedPad (SP) performance and measures from the visual search task.

		SP Simple Hits		SP Complex Hits	
Conjunction Intercept	Pearson’s r	−0.336	*	−0.301	
	df	68		68	
	*p*-value	0.032		0.088	
Conjunction Slope	Pearson’s r	−0.087		−0.11448	
	df	68		68	
	*p*-value	1.00		1.00	
Feature Intercept	Pearson’s r	−0.438	**	−0.422	**
	df	68		68	
	*p*-value	0.01		0.002	
Feature Slope	Pearson’s r	−0.158		−0.195	**
	df	68		68	
	*p*-value	1.00		0.848	

Note. * *p* < 0.05, ** *p* < 0.01. *p*-values have been adjusted for multiple comparisons using a Bonferroni correction for 8 tests.

**Table 3:** Hierarchical regression coefficients on SpeedPad Simple score.

Step 1 coefficients—SpeedPad Simple					
Predictor	Estimate	SE	t	*p*	VIF
Intercept ^a^	81.2	<0.001	<0.001	<0.001	
Exercise	10.5	<0.001	<0.001	<0.001	1.00
Step 2 coefficients—SpeedPad Simple					
Predictor	Estimate	SE	t	*p*	VIF
Intercept ^a^	90.92	2.46	36.98	<0.001	
Exercise	9.28	2.46	3.77	<0.001	1.01
Cueing Benefit	−0.026	0.05	5.84	<0.001	1.01
Step 3 coefficients—SpeedPad Simple					
Predictor	Estimate	SE	t	*p*	VIF
Intercept ^a^	107.49	8.60	12.50	<0.001	
Exercise	8.94	2.41	3.70	<0.001	1.01
Cueing Benefit	−0.22	0.05	4.43	<0.001	1.26
Visual Search Intercept Feature	−0.03	0.02	2.01	0.049	1.27

^a^ Represents reference level.

**Table 4:** Hierarchical regression coefficients on SpeedPad Complex score.

Step 1 coefficients—SpeedPad Complex					
Predictor	Estimate	SE	t	*p*	VIF
Intercept ^a^	71.31		38.53	<0.001	
Exercise	9.48	2.51	3.77	<0.001	1.00
Step 2 coefficients—SpeedPad Complex					
Predictor	Estimate	SE	t	*p*	VIF
Intercept ^a^	78.28		35.50	<0.001	
Exercise	8.59	2.21	3.89	<0.001	1.01
Cueing Benefit	−0.19	0.04	4.66	<0.001	1.01
Step 3 coefficients—SpeedPad Complex					
Predictor	Estimate	SE	t	*p*	VIF
Intercept ^a^	94.01		12.24	<0.001	
Exercise	8.26	2.16	3.83	<0.001	1.01
Cueing Benefit	−0.15	0.04	3.31	0.001	1.26
Visual Search Intercept Feature	−0.03	0.02	2.13	0.037	1.27

^a^ Represents reference level.

## Data Availability

Raw data and R scripts for preprocessing are publicly available at OSF: https://osf.io/dwmu5/. Further inquiries can be directed to the corresponding author.
